# Efficacy of diode-emitting diode (LED) photobiomodulation in pain management, facial edema, trismus, and quality of life after extraction of retained lower third molars: A randomized, double-blind, placebo-controlled clinical trial

**DOI:** 10.1097/MD.0000000000012264

**Published:** 2018-09-14

**Authors:** Carlos Alberto Tenis, Manoela Domingues Martins, Marcela Leticia Leal Gonçalves, Daniela de Fátima Teixeira da Silva, João Júlio da Cunha Filho, Marco Antonio Trevizani Martins, Raquel Agnelli Mesquita-Ferrari, Sandra Kalil Bussadori, Kristianne Porta Santos Fernandes

**Affiliations:** a Universidade Nove de Julho, Liberdade , São Paulo SP; b Universidade Federal do Rio Grande do Sul, Universidade Estadual de Campinas, Paulo Gama Avenue, Farroupilha , Porto Alegre, RS; c Universidade Nove de Julho, Liberdade, São Paulo, SP; d Pontifícia Universidade Católica do Rio Grande do Sul, Ipiranga Avenue, Paternon; e Faculty of Dentistry of Universidade Federal do Rio Grande do Sul, Paulo Gama Avenue, Farroupilha, Porto Alegre, RS; f Biophotonics Applied to Health Sciences, Universidade Nove de Julho, Liberdade; g Universidade Nove de Julho, Liberdade, São Paulo, SP, Brazil.

**Keywords:** edema, LED, pain, photobiomodulation, randomized clinical trial, tooth extraction

## Abstract

**Background:**

In dentistry, one of the most common surgical procedures is the removal of retained third molars. This surgery generates great morbidity to the participants for causing pain, edema, and trismus due to surgical trauma. The objective of the present study is to evaluate the efficacy of photobiomodulation with light emitting diode (LED) in the control of pain, facial edema, trismus, and quality of life resulting from the extraction of retained lower third molars.

**Methods:**

A randomized, double-blind, placebo-controlled clinical trial involving 38 adult participants, who meet the criteria of eligibility and agree to participate in the study. Before the surgeries are performed, the facial and mouth opening measures of all the participants will be taken. Immediately after the surgeries, participants will be randomized into 2 groups. In the LED group, participants will receive LED applications (intra oral with 660 nm, 12J and extraoral with 850 nm, 108J) in the immediate postoperative, first and second days after the surgical procedure. In the control group, the participants will be attended in the same way as in the LED group, however, the person in charge of the application will simulate the irradiation. Pain (EVA and NRS-101), postoperative edema, trismus, temperature, dysphagia, and hematoma will be evaluated after 1, 2, 5, and 7 days. The oral health impact profile (OHIP-14 Questionnaire) and anxiety analysis (Beck anxiety inventory -BAI) questionnaires will be applied preoperatively and 7 days after treatment. The appropriate statistical tests will be applied for each specific analysis in a significance level of 5%.

**Discussion:**

Although the use of low-power laser in the postoperative has shown good results in the control of postoperative sequelae, this is the first study on the efficacy of the use of LED in this situation.

## 1 Introduction

Photobiomodulation (PBM) is an emerging technique using red or near infrared light (low-power laser or light emitting diode [LED]) to modulate inflammation, accelerate wound healing, and reduce pain and discomfort in different clinical situations.^[1,2]^ The efficacy of PBM depends on the parameters, such as wavelength, power output, and energy. Different clinical situations require different PBM protocols. Most of the research on PBM in dentistry was carried out with low power/intensity lasers and some protocols are already well established using these lasers.^[2]^ However, few studies in dentistry were developed using LEDs, and most of them were performed to prevent/treat oral mucositis.^[3]^

Some studies have demonstrated a beneficial effect of LED (670 nm) on the incidence and severity of oral mucositis in participants undergoing oncological treatment.^[4–6]^ However, the near-infrared light in the 850 nm range has been shown to be capable of promoting vasodilation and the production of growth factors, as well as angiogenesis, leading to wound healing.^[7,8]^

In dentistry, one of the most common surgical procedures is the removal of retained third molars.^[2,9]^ This surgery generates great morbidity to the participants by causing pain, edema, and trismus due to surgical trauma.^[2,10,11]^ In recent years, several studies have shown beneficial effects of low intensity laser therapy in the postoperative period of lower third molar extractions.^[12–14]^. Despite the beneficial effects of LEDs on pain control and repair described in the literature, no studies were found to evaluate the effect of the use of LEDs in the postoperative period of third molar surgery. This is a relatively new technology and it is still under investigation especially in clinical trials.

The objective of the present study is to evaluate the efficacy of LED photobiomodulation in pain control, facial edema, trismus, temperature, dysphagia, hematomas, and quality of life after the extraction of retained lower third molars.

## 2 Methods/design

### 2.1 Type of study

A randomized, double-blind, placebo-controlled clinical trial will be conducted to determine the efficacy of diode-emitting diode (LED) PBM on the adverse effects of extraction of retained lower third molars. The study will be conducted at the Faculty of Dentistry of UFRGS, Department of Surgery and Orthopedics (DCO), from May 2018 to May 2019.

The study will be conducted in accordance with the guidelines of good clinical practice and has been approved by the Research Ethics Committee of UFRGS under process number 82570818.0.0000.5347. It follows the 466/2012 resolution of the National Health Council. After clarification and authorization of participants (or their guardians, for those under age) they shall sign a Free and Informed Consent Form.

**Figure 1:**
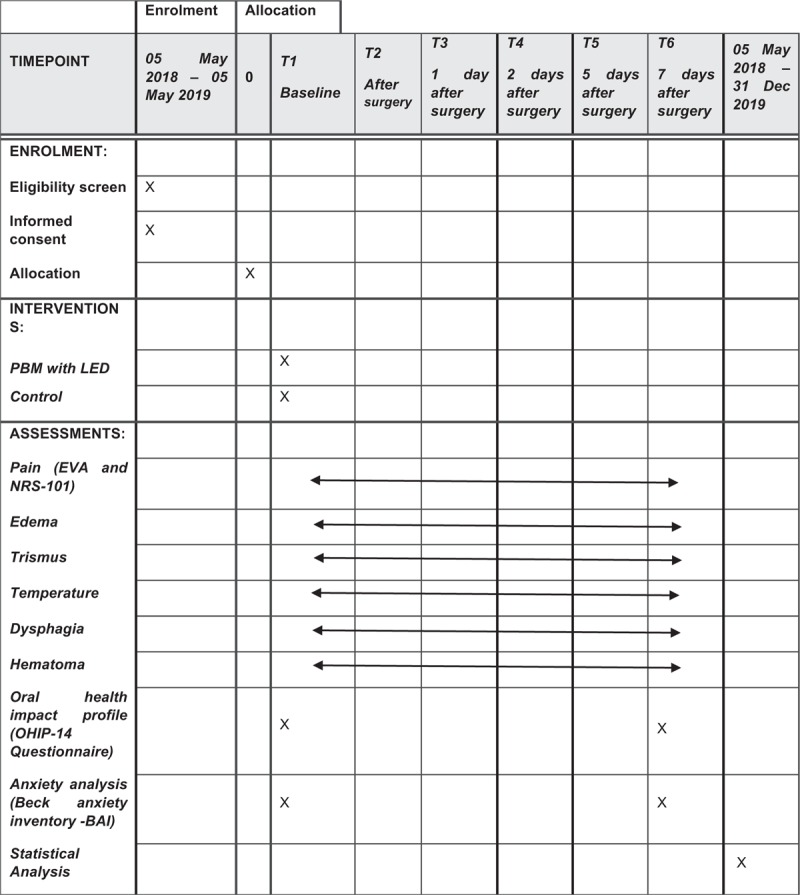
SPIRIT figure as recommended by 2013 SPIRIT Statement.

The protocol is in accordance with the 2013 SPIRIT (Standard Protocol Items: Recommendations for Interventional Trials) Statement. The SPIRIT checklist can be found as an additional file and Figure 1 is the SPIRIT figure. SPIRIT was developed to provide guidance in the form of a checklist of recommended items to include in a clinical trial protocol, to help improve its content and quality.

### 2.2 Trial registration

Clinicaltrials.gov as NCT03442166, first posted February 22, 2018 and last updated May 17, 2018; https://clinicaltrials.gov/ct2/show/NCT03442166

### 2.3 Sample calculation

A sample calculation based on the parameters of variability of the article of Eshghpour, Ahrari ^[13]^ based on the pain analysis of the participants on the 3rd postoperative day evaluated by a visual analog scale (VAS) was performed. Using a power of 80% and level of significance of 5%, a minimum sample size of 17 cases per group was obtained, making a total of 34 participants for the study. Winpepi software version 10.5 was used for this calculation. Considering a loss of 10%, based on the study that was used for the sample calculation, a loss of 2 participants per group should be predicted. Therefore, the total number of participants to be selected will be 38 individuals.

*Inclusion criteria*:

- Need of surgical removal of retained lower third molars;
- Agree to participate in the study after reading and signing the Informed Consent Term;
- Participants with indication for extraction of lower third molars (recurrent infections, bad position, and orthodontic indication) or professional written indication, healthy patients (negative medical history), systolic blood pressure lower than 140 mm Hg, and diastolic blood pressure lower than 90 mm Hg and heart rate values of 70 ± 20 beats/minute. The upper and lower central incisor teeth must be present.

*Exclusion criteria*:

- Systemic diseases, chronic pain or neurological and psychiatric disorders;
- Smokers;
- Using anti-inflammatories, analgesics or bisphosphonates in the last 15 days;
- Present active pericoronarite;
- Pregnant;
- Breastfeeding;
- Severe temporomandibular disorders;
- Photosensitivity history;
- Allergic to any drug used in the research (paracetamol, chlorhexidine 2%);
- Participants presenting radiolucent images associated with the teeth to be extracted;
- Participants who present any type of complication during surgery (hemorrhage, operative difficulty, time greater than 90 minutes of surgery), as these cases are not in the standard expected for third molar surgeries. In this case, the central action analgesic will be prescribed. These data will not be part of the statistical analysis but will be described and discussed.

### 2.4 Recruitment and randomization

Thirty-eight participants of both genders referred to the Faculty of Dentistry of UFRGS who need to perform the removal of lower third molars will be selected. Participants will be approached by means of an oral invitation to be conducted after verifying the eligibility criteria of the subject for the research.

To randomly distribute the subjects in the 2 experimental groups, a draw will be made with numbers. As the numbers are drawn, they will compose the experimental groups. Opaque envelopes will be identified with each number and inside it a sheet containing the information of the corresponding experimental group will be inserted according to the order obtained in the draw. The envelopes will be sealed and will remain sealed in numerical order in a safe place until the time of the surgeries. The drawing and preparation of the envelopes will be performed by a person who is not involved in the study.

Participants will be evaluated by the surgeon and when they meet all eligibility criteria previously described, they will be included in the study. All will be submitted to the same surgical protocol. Immediately after the surgeries, the researcher responsible for applying the LED will remove and open one envelope (without changing the numerical sequence of the other envelopes) and perform the indicated procedure.

The 38 participants will be allocated in the experimental groups as follows:

LED group (n = 19): Participants will receive 3 applications of intraoral LEDs with 660 nm and extraoral with 850 nm (Oncollux, Cosmedical, São Paulo, SP, Brazil) in the immediate postoperative, first and second days after the surgical procedure. The intraoral irradiation will be performed with a 6 red LED cluster and in the extraoral site a 36 infrared LED cluster will be used. The parameters of red/infrared LED are describe in Table 1.

**Table 1:**
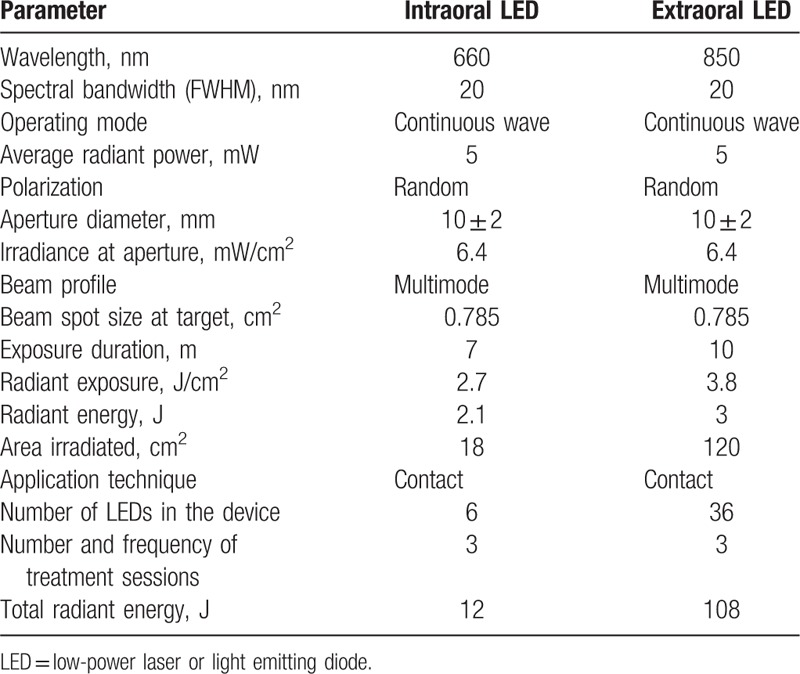
LED parameters.

Control group (n = 19)—Participants will be attended in the same way as the LED group. The person in charge of the application will simulate the intraoral and extraoral irradiation by positioning the LED in the same locations described for the LED group, but the equipment will be kept off. So that the patient does not identify the sound of activation of the device (beep), it will be recorded, and connected at the time of application.

## 3 Evaluations

### 3.1 Preoperative assessment

#### 3.1.1 Facial measures

Measurements of the distances between the corner of the eye and the angle of the mandible, between the tragus and the labial commissure, and between the tragus and the pogonium of each patient will be taken before the surgical procedure.

#### 3.1.2 Mouth opening

The opening of the mouth will be measured by the distance between the incisal edges of the upper and lower central incisors using a 150 mm hand-operated caliper (ECCOFER, Curitiba, Paraná, Brazil) as previously described.^[15,16]^

### 3.2 Surgery

The procedures will be performed by a surgeon specializing in maxillofacial surgery. The surgical material and the operative technique will be determined according to protocol for removal surgeries of retained third molars from School of Dentistry of UFRGS. The following procedures will be performed in each surgery: extra and intraoral antisepsis, infiltrative anesthesia by block and infiltrative terminal of the tooth and attached structures, isolation and drying of the operative field with gauze, incision with proximal extension of the teeth involved in order to allow access to the exposure surgical removal of the gingival flap, osteotomy for access (when necessary), muco-periosteal detachment, ostectomy, odontosection with electric motors (when necessary), aspiration of blood and liquids used for irrigation and washing of the operative field, detachment and removal of the gingival flap, avulsion of the dental element, irrigation and suture.

All participants will receive the following medications: Codeine 30 mg, paracetamol 500 mg to be used every 6 hours, in the first 24 hours. Then, they will use paracetamol 750 mg every 6 hours for the duration of the painful symptomatology. They will be asked for information on the amount of medicine ingested. The data of these participants will only be described, but not included in the analysis of the results. Such exclusion shall not prejudice the treatment.

### 3.3 Evaluation of the surgical procedure by the surgeon

At the end of the surgeries, the maxillofacial surgeon will record the following: the position of the retained tooth, the degree of difficulty of the procedure, the number of anesthesia tubes used, the occurrence of hemorrhage during surgery and during suturing and the duration of the surgery (from the first incision to the end of the suture).

The position of the included tooth will be determined based on the classifications proposed by Winter^[17]^ and by Pell and Gregory.^[18]^

The degree of difficulty of the surgery will be evaluated by the Prant scale modified by Amarillas-Escobar et al,^[19]^ which classifies the surgical procedure as follows: grade 1=extraction with only a forceps; grade 2=extraction with osteotomy; grade 3=extraction with osteotomy and coronal section; and grade 4=complex extraction.

### 3.4 Procedures to guarantee double blinding in the postoperative period

After the suture is completed, the researcher responsible for applying the LED will open the envelope containing the information from the experimental group in which the patient will be inserted and proceed to the experiment. A single examiner will run the LED application and will not perform any type of evaluation. Surgical procedures will be performed by the same surgeon. The preoperative and postoperative evaluations (1, 2, 5, and 7 days postoperatively) will be done by an examiner who will not be aware of the group in which each patient is allocated. The information that will be obtained in the evaluations will be written in the evaluator's file. Participants will not be aware of whether or not they received LED irradiation because the person responsible for applying the LED will position the insert in the irradiation sites in all participants and will only trigger light when and where predicted in the specific experimental group. The characteristic sound of the device will be triggered by recording in the control group.

### 3.5 Postoperative pain evaluation

In this project, we intend to apply 2 scales: the VAS and NRS-101. The VAS will be printed on the patient evaluation form and the subjects will be instructed by the evaluator to mark a point on the 10 cm line, indicating the intensity of their pain after 1.2, 5, and 7 days of the surgeries For the NRS-101 scale, the evaluators will ask participants to assign a number between 0 (no pain) and 100 (worst possible pain) that best represents the pain they are currently experiencing after 1, 2, 5, and 7 days of surgeries.

### 3.6 Evaluation of postoperative edema

The evaluator will measure the distances between the corner of the eye and angle of the mandible, between tragus and labial commissure, and between tragus and pogonium^[14]^ of each patient 1, 2, 5, and 7 days after surgery.

### 3.7 Postoperative muscle spasm assessment

This result is usually evaluated by measuring the distance between the incisal edges of the upper and lower central incisors, using a caliper rule. In the present study, the evaluator will measure the opening of the mouth in each patient 1, 2, 5 and 7 days after surgery.

### 3.8 Temperature

The temperature will be measured locally and systemically in each patient 1, 2, 5, and 7 days after surgery. The local measurement will be done with a digital thermometer (Safety 1st, “No Touch Forehead”, Columbus), in the region of the mandibular angle, 2 cm above the lower border of the mandible and 3 cm for mesial of the branch of the mandible, both on the operated side and on the opposite side. The systemic temperature will be measured in the frontal region of the patient in the median position 3 cm above the glabella in the same time frames.

### 3.9 Dysphagia

The evaluation of dysphagia will be performed through a numerical scale in which: (0) total absence of dysphagia; (1) dysphagia to solid foods; (2) dysphagia to any liquid or solid food. The patients will respond to the questioning on days 1, 2, 5, and 7 days after surgery.

### 3.10 Evaluation of the presence and intensity of hematoma/bruise

The presence of hematoma/ecchymosis will be evaluated by measuring the largest diameter of colorimetric changes in the skin of the jugal and submandibular region at 1, 2, 5, and 7 days after surgery. The measure will be performed by the evaluator who will classify the occurrence of this result into 4 categories: none; larger diameter smaller than 4 cm; greater diameter between 4 and 10 cm, and larger diameter >10 cm.

### 3.11 Analysis of the oral health impact profile (OHIP-14 questionnaire)

The oral health impact profile (OHIP-14) is a simplified form of the original OHIP questionnaire that is used to assess the impact of oral health on subjects’ quality of life. The items are distributed among the following subscales: functional limitation, pain, psychological discomfort, physical disability, psychological deficiency, social incapacity and disability. The questionnaire will be applied by the evaluator in the preoperative period and 7 days after surgery.

### 3.12 Anxiety analysis

The analysis of anxiety will be done through Beck's inventory of anxiety that evaluates, by quantitative approximation, anxiety symptoms. The questionnaire contains 21 aspects that reflect somatically, cognitively and affectively the characteristic symptoms of anxiety. The questionnaire also presents high reliability, high internal consistency and moderate validity, both for psychiatric participants and for general population samples.^[20]^ The inventory consists of 21 items that are descriptive statements of the symptoms common in anxiety pictures and that should be evaluated by the individual with reference to himself in the period of one week in a scale of 4 points that reflect levels of increasing severity of each symptom, whose alternatives are: absolutely not; lightly: It did not bother me much; moderately: It was very unpleasant, but I could bear it; seriously: I could hardly bear it. At the end, the items are summed and the total score can vary from 0 to 63. All participants will respond to this questionnaire in the preoperative period and at 7 days postoperatively.

### 3.13 Statistical analysis

Initial descriptive analyses will be performed considering all variables measured in the study, both quantitative (mean and standard deviation) and qualitative (frequencies and percentages). Later, the appropriate statistical tests will be applied for each specific analysis. In all tests, the significance level of 5% probability or the corresponding p-value will be adopted. All analyses will be performed using the statistical software SAS for Windows, version 9.1.3.

## 4 Discussion

The removal of retained third molars is a very common procedure that causes pain, edema, and trismus due to surgical trauma. Although the use of low-power laser in the postoperative has shown excellent results in the control of postoperative sequelae,^[12–14]^ this is the first study on the efficacy of the use of LED in this situation. Besides this, recently it was suggested that the accurate combination of irradiation site and PBM wavelength could optimize the postoperative results of this therapy after third molar removal surgeries. The best match would be the use of intraoral red light and extraoral infrared light.^[15]^ This study will evaluate whether the combination of 2 wavelength LEDs used intra and extraorally will bring benefits to the post-operative of third molar removal surgeries.

## 5 Declarations

### 5.1 Ethics committee

The study will be conducted in accordance with the guidelines of good clinical practice and has been approved by the Research Ethics Committee of UFRGS under process number 82570818.0.0000.5347. It follows the 466/2012 resolution of the National Health Council. After clarification and authorization of participants (or their guardians, for those under age) they shall sign a Free and Informed Consent Form. The identity of all individuals will be preserved in all stages of the research. Changes in the study will be reported to the committee. This will guarantee the confidentiality of each patient's data. The individual who does not attend the scheduled session will be excluded from the study.

### 5.2 Data collection methods

The authors were previously trained to collect data and perform the surgeries. All authors are qualified in photobiomodulation therapy. All data will be entered electronically. The participants’ files will be stored in numerical order in a safe place and accessible only to the authors of this study.

### 5.3 Discontinuing intervention

The application of LEDs offers minimal risk, however, if during treatment, some participants report increased pain or display an infectious condition, measures such as the use of analgesics and/or antibiotics will be adopted according to clinical tolerance and individual assessment. These participants will be excluded from the study and will be followed up by the surgical team until resolution of the condition.

### 5.4 Availability of data and materials

All information collected from the participants will be transcribed into a database replacing the individuals’ names with the registration number of the evaluation form. The datasets generated and analysed during the present study are available from the corresponding author at reasonable request. After the analysis of the data, volunteers will be invited to a meeting and the results will be shared and they will become public.

## Author contributions

Conceive and design the study: KPSF, SKB, MDM; will perform the experiment: CAT, JJCF, MATM; will analyze the data: MLLG, DFTS, RAMF, KPSF, SKB; will perform the statistical analysis: DFTS, RAMF; write the paper: CAT, MDM, MLLG, KPSF.

**Conceptualization:** Manoela Domingues Martins, Sandra Kalil Bussadori, Kristianne Porta Santos Fernandes.

**Data curation:** Marcela Leticia Leal Gonçalves, Daniela de Fátima Teixeira da Silva, Sandra Kalil Bussadori.

**Formal analysis:** Marcela Leticia Leal Gonçalves, Daniela de Fátima Teixeira da Silva, Raquel Agnelli Mesquita-Ferrari, Sandra Kalil Bussadori, Kristianne Porta Santos Fernandes.

**Investigation:** Carlos Alberto Tenis, João Julho da Cunha Filho, Marco Antonio Trevizani Martins.

**Methodology:** Carlos Alberto Tenis, João Julho da Cunha Filho, Marco Antonio Trevizani Martins.

**Software:** Daniela de Fátima Teixeira da Silva.

**Supervision:** Kristianne Porta Santos Fernandes.

**Writing – original draft:** Carlos Alberto Tenis.

**Writing – review & editing:** Manoela Domingues Martins, Marcela Leticia Leal Gonçalves, Kristianne Porta Santos Fernandes.

## References

[1] Hamblin MR Mechanisms and applications of the anti-inflammatory effects of photobiomodulation. AIMS Biophys 2017;4:337–61. 28748217 10.3934/biophy.2017.3.337 PMC5523874

[2] Brignardello-Petersen R Laser use may improve pain and wound healing in patients with recurrent aphtous stomatitis. J Am Dent Assoc 2017;148:e112. 28688511 10.1016/j.adaj.2017.05.025

[3] Campos L Cruz ÉP Pereira FS Comparative study among three different phototherapy protocols to treat chemotherapy-induced oral mucositis in hamsters. J Biophotonics 2016;9:1236–45. 27105906 10.1002/jbio.201600014

[4] Almeida Issa MC Piñeiro-Maceira J Farias RE Immunohistochemical expression of matrix metalloproteinases in photodamaged skin by photodynamic therapy. Br J Dermatol 2009;161:647–53. 19519826 10.1111/j.1365-2133.2009.09326.x

[5] Barolet D Roberge CJ Auger FA Regulation of skin collagen metabolism in vitro using a pulsed 660 nm LED light source: clinical correlation with a single-blinded study. J Invest Dermatol 2009;129:2751–9. 19587693 10.1038/jid.2009.186

[6] Corti L Chiarion-Sileni V Aversa S Treatment of chemotherapy-induced oral mucositis with light-emitting diode. Photomed Laser Surg 2006;24:207–13. 16706701 10.1089/pho.2006.24.207

[7] Hunter S Langemo D Hanson D The use of monochromatic infrared energy in wound management. Adv Skin Wound Care 2007;20:265–6. 17473561 10.1097/01.ASW.0000269312.45886.00

[8] Opel DR Hagstrom E Pace AK Light-emitting diodes: a brief review and clinical experience. J Clin Aesthet Dermatol 2015;8:36–44. PMC4479368 26155326

[9] Grossi GB Maiorana C Garramone RA Assessing postoperative discomfort after third molar surgery: a prospective study. J Oral Maxillofac Surg 2007;65:901–17. 17448840 10.1016/j.joms.2005.12.046

[10] Bello SA Adeyemo WL Bamgbose BO Effect of age, impaction types and operative time on inflammatory tissue reactions following lower third molar surgery. Head Face Med 2011;7:8. 21527036 10.1186/1746-160X-7-8 PMC3114767

[11] Sato FR Asprino L de Araújo DE Short-term outcome of postoperative patient recovery perception after surgical removal of third molars. J Oral Maxillofac Surg 2009;67:1083–91. 19375022 10.1016/j.joms.2008.09.032

[12] Alan H Yolcu Ü Koparal M Evaluation of the effects of the low-level laser therapy on swelling, pain, and trismus after removal of impacted lower third molar. Head Face Med 2016;12:25. 27457369 10.1186/s13005-016-0121-1 PMC4960798

[13] Eshghpour M Ahrari F Takallu M Is low-level laser therapy effective in the management of pain and swelling after mandibular third molar surgery? J Oral Maxillofac Surg 2016;74:1322.e1e1–8. 27055228 10.1016/j.joms.2016.02.030

[14] Sierra SO Deana AM Bussadori SK Effect of low-intensity laser treatment on pain after extraction of impacted mandibular third molars: a randomised, controlled, clinical trial. Br J Oral Maxillofac Surg 2015;53:996–1000. 26421525 10.1016/j.bjoms.2015.09.006

[15] Sierra SO Deana AM Bussadori SK Choosing between intraoral or extraoral, red or infrared laser irradiation after impacted third molar extraction. Lasers Surg Med 2016;48:511–8. 26868520 10.1002/lsm.22488

[16] Aras MH Gungormus M The effect of low-level laser therapy on trismus and facial swelling following surgical extraction of a lower third molar. Photomed Laser Surg 2009;27:21–4. 19196113 10.1089/pho.2008.2258

[17] Winter GB Impacted Mandibular Third Molar. St. Louis: American Medical Book; 1926.

[18] Pell GJ Gregory BT Impacted mandibular third molars classification and modified technique for removal. Dental Dig 1933;39:330–8.

[19] Amarillas-Escobar ED Toranzo-Fernández JM Martínez-Rider R Use of therapeutic laser after surgical removal of impacted lower third molars. J Oral Maxillofac Surg 2010;68:319–24. 20116702 10.1016/j.joms.2009.07.037

[20] Volpato LE de Oliveira RC Espinosa MM Viability of fibroblasts cultured under nutritional stress irradiated with red laser, infrared laser, and red light-emitting diode. J Biomed Opt 2011;16:075004. 21806261 10.1117/1.3602850

