# Efficacy of diode-emitting diode (LED) photobiomodulation in pain management, facial edema, trismus, and quality of life after extraction of retained lower third molars: A randomized, double-blind, placebo-controlled clinical trial: Erratum

**DOI:** 10.1097/MD.0000000000012882

**Published:** 2018-10-12

In the article, “Efficacy of diode-emitting diode (LED) photobiomodulation in pain management, facial edema, trismus, and quality of life after extraction of retained lower third molars: A randomized, double-blind, placebo-controlled clinical trial”,^[1]^ which appears in Volume 97, Issue 37 of *Medicine*, the title of the article should be “Efficacy of light-emitting diode (LED) photobiomodulation in pain management, facial edema, trismus, and quality of life after extraction of retained lower third molars: A randomized, double-blind, placebo-controlled clinical trial.”
